# Thanks to Reviewers 2021

**DOI:** 10.5334/pb.1149

**Published:** 2022-01-18

**Authors:** Editorial Team

**Affiliations:** 1 Psychologica Belgica, BE

## Abstract

All manuscripts published in Psychologica Belgica have been assessed conscientiously and unselfishly by expert reviewers. The quality of our journal totally depends on their valuable and constructive criticisms to the authors. Both the editors and the authors highly appreciate the input and dedication of all our reviewers. Many thanks.

Cristina Antunes

Lucie Attout

Audrey Babic

Paula Barata

Christine Bastin

Tom Beckers

Eva Billen

Joël Billieux

Adelaide Blavier

Anke Boone

Guillermo Borragan Pedraz

Badiaa Bouazzaoui

Lieven Brebels

Poppy Brown

Olivier Bruyère

Yohanes Budiarto

Andreea Butucescu

Jeroen Camps

Arnaud Carre

Fabienne Collette

Christian Crandall

Catherine Culot

Rudi D’Hooge

Kim de Jong

Hans De Witte

Egon Dejonckheere

Ann DeSmet

Sandrine Detandt

Hazal Duman Alptekin

Emir Efendic

Jonas Everaert

Louis Favril

Chiara Fini

Erik Franck

Courcy François

Sarah Galdiolo

Amy Gentzler

Robin Gerrits

Fabien Gierski

Nicolas Gillet

Klaske Glashouwer

Cassandra Gois

Philippe Golay

Brice Gouvernet

Loris Grandjean

Michel Hansenne

Marie Helweg-Larsen

Joeri Hofmans

Vera Hoorens

Typhaine Huyghebaert-Zouaghi

Mareike Kaemmerer

Julien Laloyaux

Emilie Lapointe

Patrick Lemaire

Christophe Leys

Audrey Linder

Olivier Luminet

Patrick Luyten

Rich McNally

Miriam Metzger

Isabelle Milhabet

Cindy Mottrie

Santos Orejudo

Jordi Ortet Walker

Javier Ortuño Sierra

Danny Osborne

Behcet Ozkara

Anne-Lise Pitel

Karolien Poels

Koen Ponnet

Igor Portoghese

Chuntana Reangsing

David D. Ritchie

Arne Roets

Ralph Erich Schmidt

Janneke M. Schokkenbroek

Ralf Schulze

James Shepperd

Jessica Simon

Mieke Slim

Arnaud Szmalec

Chloé Tolmatcheff

Sébastien Urben

Stephan Van den Broucke

Nele Van den Cruyce

Joris van Loenhout

Kathleen Van Royen

Christian Vandenberghe

Audrey Vicenzutto

Cato Waerloos

Sylvie Willems

Marlis Wullenkord

